# Prolidase Deficiency Causes Spontaneous T Cell Activation and Lupus-like Autoimmunity

**DOI:** 10.4049/jimmunol.2200212

**Published:** 2023-01-11

**Authors:** Rose Hodgson, Tanya L. Crockford, Aneesha Bhandari, Jessica D. Kepple, Jennifer Back, Eleanor Cawthorne, Lucie Abeler-Dörner, Adam G. Laing, Simon Clare, Anneliese Speak, David J. Adams, Gordon Dougan, Adrian C. Hayday, Mukta Deobagkar-Lele, Richard J. Cornall, Katherine R. Bull

**Affiliations:** * MRC Human Immunology Unit, Nuffield Department of Medicine, University of Oxford, Oxford, United Kingdom;; † Department of Immunobiology, King’s College London, London, United Kingdom;; ‡ The Francis Crick Institute, London, United Kingdom; and; § Wellcome Sanger Institute, Hinxton, United Kingdom

## Abstract

Prolidase deficiency (PD) is a multisystem disorder caused by mutations in the *PEPD* gene, which encodes a ubiquitously expressed metallopeptidase essential for the hydrolysis of dipeptides containing C-terminal proline or hydroxyproline. PD typically presents in childhood with developmental delay, skin ulcers, recurrent infections, and, in some patients, autoimmune features that can mimic systemic lupus erythematosus. The basis for the autoimmune association is uncertain, but might be due to self-antigen exposure with tissue damage, or indirectly driven by chronic infection and microbial burden. In this study, we address the question of causation and show that *Pepd*-null mice have increased antinuclear autoantibodies and raised serum IgA, accompanied by kidney immune complex deposition, consistent with a systemic lupus erythematosus–like disease. These features are associated with an accumulation of CD4 and CD8 effector T cells in the spleen and liver. *Pepd* deficiency leads to spontaneous T cell activation and proliferation into the effector subset, which is cell intrinsic and independent of Ag receptor specificity or antigenic stimulation. However, an increase in KLRG1^+^ effector CD8 cells is not observed in mixed chimeras, in which the autoimmune phenotype is also absent. Our findings link autoimmune susceptibility in PD to spontaneous T cell dysfunction, likely to be acting in combination with immune activators that lie outside the hemopoietic system but result from the abnormal metabolism or loss of nonenzymatic prolidase function. This knowledge provides insight into the role of prolidase in the maintenance of self-tolerance and highlights the importance of treatment to control T cell activation.

## Key Points

- We demonstrate a causal link between prolidase deficiency and autoimmunity.
- Loss of PEPD leads to an Ag-independent increase in effector CD8 T cells.
- Cell-extrinsic expansion of KLRG1^+^ CD8 effectors is associated with PEPD autoimmunity.

## Introduction

Prolidase is a ubiquitously expressed metallopeptidase that is uniquely required for the hydrolysis of dipeptides containing C-terminal proline or hydroxyproline, and hence the breakdown of proline-rich substrates including collagen (1). Prolidase deficiency (PD) is rare, with an incidence estimated at 1 in 1 million births (2). Individuals with severely diminished or absent prolidase activity have imidodipeptiduria and may exhibit a wide range of clinical features, including facial dysmorphism, developmental delay, lower limb skin ulceration, anemia, and recurrent respiratory infections (3–7).

Curiously, autoimmune and inflammatory features are also common in PD patients (8–11), including hypergammaglobulinemia with high titers of IgE and IgA, positivity for antinuclear Abs (ANAs) (21%), and splenomegaly (45%). Fifteen percent of PD patients are diagnosed with an autoimmune disorder, and 6% meet the formal diagnostic criteria for systemic lupus erythematosus (SLE) (7). However, it is unclear whether the loss of immunological tolerance is triggered by direct effects of PD on immune function or by exposure to self-antigens in the context of chronic skin or respiratory infections.

To address these questions in a physiologically relevant setting, we characterized *Pepd*-null *(Pepd*^−/−^) mice that were generated as part of the United Kingdom–wide Infection, Immunity and Immunophenotyping consortium (12). *Pepd*^−/−^ mice have autoimmune disease associated with a higher incidence of class-switched ANAs than do wild-type (WT) controls, raised serum IgA as seen in PD patients, and glomerular Ig deposition. We show that prolidase is a cell-intrinsic regulator of CD4 and CD8 effector T cell expansion, revealing a role for prolidase-mediated T cell regulation in self-tolerance.

## Materials and Methods

### Animal work

All procedures were performed in accordance with the Animals (Scientific Procedures) Act 1986, amended 2012, with procedures reviewed by the clinical medicine Animal Welfare and Ethical Review Body and conducted under Home Office Project License P79A4C5BA. Mice were bred in specific pathogen-free individually ventilated cages at the Wellcome Trust Centre for Human Genomics. The only reported positives on Federation of European Laboratory Animal Science Associations health screening during the entire time course of these studies were for *Helicobacter* spp., *Chilomastix* spp., *Enteromonas muris*, *Trichomonas* spp., mouse norovirus, and *Entamoeba* spp. Experimental animals were not excluded from analysis except according to prespecified experimental design on the basis of failed chimeric reconstitution or failed cell transfer. All experiments included age- and sex-matched control animals, and controls were cohoused littermates wherever possible.

C57BL/6JOlaHsd mice were purchased from Envigo. B6SJL-CD45.1(Ly5^a^) mice were bred at the Department of Biomedical Services, Oxford University (Oxford, U.K.). OT-I mice (C57BL/6-Tg(TcraTcrb)1100Mjb/J) have been described previously. *Pepd* mice (*Pepd* ^tm1a(KOMP)Wtsi^) have been previously described (12) and were ordered from the Knockout Mouse Programme/International Mouse Phenotyping Consortium. Positive control mice for lupus-like phenotype screening were MRL/MpJ-*Fas^lpr^*/J, ordered from The Jackson Laboratory (strain 000485), and BM12 mice, also from The Jackson Laboratory (strain 001162) (13). Genotyping of *Pepd* mutant mice was performed by PCR or by Sanger sequencing using the following primers: *Pepd* forward, 5′-CCCAGGCTATCTCAAAGCTACG-3′ and *Pepd* reverse, 5′-AATGGCTCCAAGACCCACAG-3′ and 5′-TCGTGGTATCGTTATGCGCC-3′.

### Irradiation bone marrow chimeras

CD45-1^+^ heterozygous or homozygous female recipients were irradiated with two doses of 4.5 Gy, spaced by 3 h and then i.v. injected with at least 5 × 10^6^ donor bone marrow (BM) cells taken from female donor mice. BM chimeras were allowed to reconstitute for at least 8 wk before immunization or analysis.

### BrdU incorporation

Specified mice received BrdU for 7 d in their drinking water at a final concentration of 0.8 mg/ml.

### Immunization and recall experiments

For modified vaccinia virus Ankara (MVA) immunization, mice were anesthetized and immunized intradermally or i.m. with MVA expressing OVA at a dose of 5 × 10^6^ PFU/mouse. Ag-specific cells in the peripheral lymphoid organs were identified by ex vivo epitope stimulation, and cytokine production was measured as described previously (14).

### Magnetic cell sorting

Splenic total T cells were isolated using magnetic sorting and a Miltenyi Biotec pan T cell isolation kit II (no. 130-095-130).

### Flow cytometry

Isolated cell suspensions were stained for flow cytometric analysis as previously described (15), and data were collected on a BD FACSCanto (10-color). The following flow cytometry Abs were from BioLegend: anti-IgD (nos. 405704, 405716, 405708), anti-IgM (nos. 406508, 406512, 406514), anti-CD45.1 (nos. 110708, 110716, 110730, 110722), anti-CD45.2 (nos. 109841, 109824, 109818, 109832), anti-CD23 (no. 101614), anti-CD93 (no. 136510), anti-CD19 (nos. 115528, 115530), anti-CD3 (nos. 100330, 100328, 100214), Zombie Aqua live/dead (no. 423102), near-infrared live/dead (no. 423106), anti-B220 (nos. 103236, 103232, 103212, 103243), anti-CD21 (no. 123418), anti-CD24 (no. 101836), anti-CD25 (nos. 102008, 102022), anti-CD44 (nos. 103006, 103012, 103020), anti-CD62L (nos. 104406, 104408, 104412), anti-PD1 (no. 109110), anti-CD4 (nos. 100528, 100430, 100414), anti-CD8a (nos. 100734, 100730, 100714), anti-TCR Vα2 (no. 127806), anti–Ki-67 (nos. 652408, 652422), anti-Ly6C (no. 128012), anti-KLRG1 (no. 138408), anti-Foxp3 (no. 126404), and anti–IFN-γ (no. 505808). The following flow cytometry Abs were from eBioscience: anti-IgM (no. 48-5890-82), anti-PD1 (no. 11-9981-82), and anti–IFN-γ (nos. 48-7311-82, 25-7311-82). The following flow cytometry Abs were from BD Pharmingen: anti-CD43 (no. 562865), anti-CD21 (no. 563176), anti–BP-1 (no. 553735), anti-IgA (no. 559354), anti-Bcl2 (no. 556537), anti-Vβ5 (no. 553189), and anti-BrdU (no. 364108). Anti-CD44 (no. 45-0441-82), CellTrace Violet proliferation dye (no. C34557), and anti-CD4 (no. 25-0041-82) were purchased from Life Technologies. Anti-Bim (no. 10408S) was purchased from CST/NEB.

### Intracellular staining

Splenocytes (1–5 × 10^6^) were fixed and permeabilized using the BD Cytofix/Cytoperm kit in combination with Perm/Wash buffer (BD Biosciences, nos. 554655, 554722, 557885) before staining with intracellular Ab.

To detect BrdU incorporation, surface-stained cells fixed as above were washed and treated with BD Cytoperm Permeabilization Buffer Plus (BD Biosciences, no. 561651) by protocol, and refixed before treatment with 30 μg of DNase/10^6^ cells. Following washing, cells were incubated with fluorescent anti-BrdU and acquired.

### FACS

Live CD45.1^−^CD45.2^+^ T cells were sorted on a FACSAria Fusion cell sorter at the Wellcome Centre for Human Genetics flow cytometry facility from magnetically isolated splenic T cells stained with live/dead (allophycocyanin-Cy7, laser-filter 640–780/60-A), anti-CD45.1 (pacific blue, 405–450/50-A), anti-CD45.2 (PE, 561–582/15-A) with negative dump channel, Alexa Fluor 700 (640–730/45-A) containing anti-B220 and anti-CD19, and negative dump channel allophycocyanin (640–670/30-A) containing CD11b, MHC class II, CD11c, and CD49b.

### Single-cell RNA sequencing sample preparation

For preparation of the nonchimeric samples, 10% WT CD45.1^+^ splenic cells were spiked into one WT CD45.2^+^ and one *Pepd*^−/−^ CD45.2^+^ sample, and 10% WT CD45.1^+^CD45.2^+^ splenic cells were spiked into one WT CD45.2^+^ and one *Pepd*^−/−^ CD45.2^+^ sample. Total T cells from the four samples described above and one WT CD45.2^+^ and one *Pepd*^−/−^ CD45.2^+^ splenic sample were isolated by magnetic sorting, and then the six samples were multiplexed using BioLegend TotalSeq-B hashing Abs by protocol (B0301, no. 155831; B0302, no. 155833; B0303, no. 155835; B0304, no. 155837; B0305, no. 155839; B0306, no. 155841). Pooled hashtagged cells were labeled with TotalSeq-A Abs by protocol (anti-CD4, no. 100569; anti-CD8a, no. 100773, anti-CD279 [PD1], no. 109123; anti-CD44, no. 103063; anti-CD62L, no. 104451; anti-CD25, no. 102055; anti-CD127 [IL-7R], no. 135045; anti-ICOS, no. 313555; anti-CD69, no. 104546; anti-CD27, no. 124235; anti-CD5, no. 100637; anti-KLRG1, no. 138431; anti-CD95, no. 152614; anti-CD45.1, no. 110753; and anti-CD45.2, no. 109853). After washing, cells were loaded into the chromium controller (10x Genomics) chip and sequenced as previously described (16). Reads for TotalSeq anti-CD45.1 and anti-CD45.2 alone could not be detected, and thus the CD45.1^+^ and CD45.1^+^/CD45^+^ populations could not be separated from the CD45.2 in the nonchimeric single-cell RNA sequencing (scRNA-seq) experiment.

For the scRNA-seq analysis of chimeric T cells, magnetically isolated T cell populations from four WT:WT and four WT:*Pepd*^−/−^ BM chimeric mice were hashtagged by sample using as above, with the addition of hashtags B0307 (no. 155843) and B0308 (no. 155845). Pooled hashtagged CD45.2^+^ T cells were isolated by FACS, then labeled with the above TotalSeq-A Abs, minus anti-CD45.1 and anti-CD45.2, then sequenced as above.

### Bioinformatics analysis

Raw single-cell sequencing data were mapped and quantified with 10x Genomics cellranger count v3.1.0, using transcriptome mm10-3.0.0 for the gene expression matrix. The EmptyDrops algorithm was used, and then a Seurat object was created from the gene expression (RNA), hashtag oligo (HTO), and surface Ab-derived tag (ADT) libraries. Using Seurat v3.1.2, the HTO assay was centered log transformation normalized and samples were demultiplexed, which also removed doublets and HTO-negative cells (17, 18).

Samples were filtered based on the gene expression assay for mitochondrial genes (<7.5%) and numbers of features (>500 and <3000) and counts (<12,000). The ADT assay was centered log transformation normalized and scaled for initial dimensionality reduction and clustering using the FindNeighbors and FindClusters functions. For clustering and dimensionality reduction, the RNA assay was normalized using the sctransform function for clustering. For differential expression and visualization, log-normalized RNA counts or raw RNA counts were used. Seurat v4.0.3 or Seurat v3.9.9 was used for weighted nearest neighbor (WNN) assay integration (18), clustering, and downstream analysis. Differential expression analysis was performed either using Seurat FindMarkers or pseudobulk with muscat v1.4.0 (19). Raw data are available at Gene Expression Omnibus under accession numbers GSE221569 (https://www.ncbi.nlm.nih.gov/geo/query/acc.cgi?acc=GSE221569) and GSE221410 (https://www.ncbi.nlm.nih.gov/geo/query/acc.cgi?acc=GSE221410).

### ELISA

Bethyl Laboratories mouse IgG (no. E90-131), IgM (no. E90-101), and IgA (no. E90-103) quantification kits were used by protocol with serum titration as follows: IgG 1:4000, IgM 1:2000, IgA 1:2000 or 1:4000, developed using tetramethylbenzidine substrate (Life Technologies, no. 00-4201-56) and detected at 450 nm. Background absorbance values were subtracted from absorbance readings before interpolation to a standard curve using hyperbola (*X* as concentration).

### ANA staining

ANA titers were detected as previously described (12) in age-matched female mice between 8 and 26 wk of age, which were imaged using a Nikon wide-field TE20000U microscope (GFP channel: ×20, 400 ms). ANA testing as part of initial screening and at the second facility after rederivation of the same *Pepd*^−/−^ strain was performed using the same assay and operators. dsDNA-specific ANAs were performed using the same method, with the exception of the use of *Crithidia luciliae* slides (Bio-Rad, no. 26109).

### Tissue fixation and sectioning

Frozen tissue was embedded in OCT (CellPath, no. KMA-0100-00A), and 5-μm cryosections were cut on a Leica Ultracut UCT ultramicrotome.

### Histology

For immunostaining, cryosections were fixed with acetone, blocked in goat serum, and then incubated with anti-mouse IgG (Thermo Fisher Scientific, no. 62-6511), anti-mouse IgA (Bethyl Laboratories, A90-103A-35), or anti-mouse C1q (Hycult Biotech, no. HM1096). After washing, slides were incubated with secondary Abs when necessary, that is, anti-goat IgG Alexa Fluor 488 (Thermo Fisher Scientific, no. A-11055), and then counterstained with DAPI (Biotium, no. 40043). Slides were mounted and sealed and then imaged at ×40 magnification on a Thermo Fisher Scientific EVOS M5000.

### Lupus band detection

OTC embedded ears from mice were sectioned and stained for basement membrane IgG deposition using an anti-mouse IgG-FITC conjugated Ab (Invitrogen, no. 62-6511, 1:200 dilution in 1% BSA/PBS). Slides were mounted using Fluoromount-G mounting media (Thermo Fisher Scientific, no. 00-4958-02) with DAPI (Thermo Fisher Scientific, no. 00-4959-52). Slides were imaged using a Leica SP8 confocal microscope.

### Creatinine measurement

Clinical chemistry was performed on a Beckman Coulter AU400 semiautomated clinical chemistry analyzer by the Mary Lyon Centre’s clinical pathology service laboratory at MRC Harwell. All assays were carried out using the manufacturer’s instructions, parameter settings, and reagents.

### Statistical analysis

GraphPad Prism software was used for statistical analyses. Unpaired, two-tailed Student *t* tests were used to determine significance between groups across subsets of pooled data from independent experiments or representative numbers data, with adjustment for multiple testing by the Holm–Sidak test. Two-way ANOVAs were used for statistical comparison between groups for subsets across independent experiments for numbers analysis.

## Results

### *Pepd*^−/−^ mice replicate the autoimmune phenotypes and increased Ab production seen in PD patients

**FIGURE 1.**
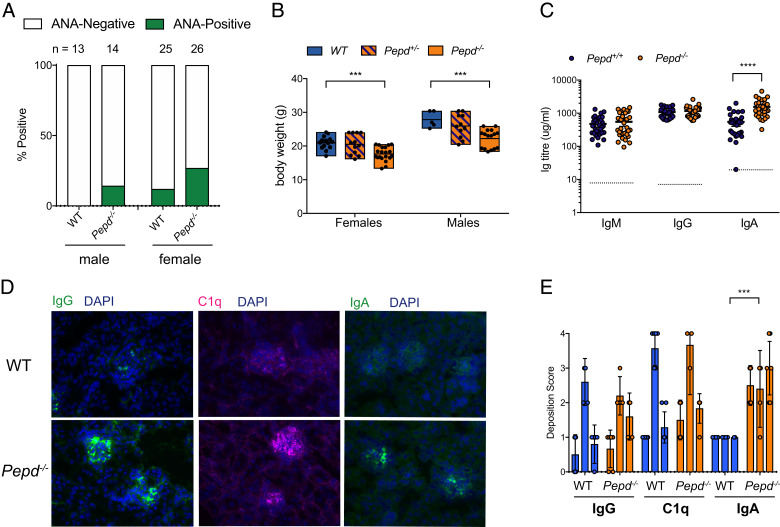
Prolidase deficiency is associated with lupus-like autoimmunity. (**A**) Percentage of samples scoring positive for antinuclear antibodies (ANAs) from WT and *Pepd*^−/−^ male and female mice age matched and between 8 and 26 wk and 12 and 40 wk, respectively, for males and females. (**B**) Weights by genotypes between 8 and 12 wk. Each point represents one mouse. For female WT versus female *Pepd*^−/−^, ***p = 0.0001; for male WT versus male *Pepd*^−/−^, ***p = 0.0006. (**C**) Serum Ig levels from WT and *Pepd*^−/−^ mice. Dotted line represents 1.5× interpolated background absorbance; each point is one mouse. ****p ≤ 0.00001. Significance for (B) and (C) was determined with an unpaired two-tailed *t* test, adjusting for multiple testing. For (A)–(C), these data are pooled from at least three independent experiments. (**D**) Representative fluorescent immunostaining at ×40 original magnification of WT and *Pepd*^−/−^ kidney sections with Abs against IgG, IgA, and C1q (green or pink), and nuclei counterstained with DAPI (blue). Images are representative of three independent experiments with two to three mice per group, sex and age matched between 9 and 16 wk. (**E**) Immunofluorescence kidney images were blinded and scored by eye by an independent experimenter and then averaged across at least three images per mouse at ×40 original magnification. Significance was determined by two-way ANOVA across experiments. Each point is one mouse, and these data are representative of three independent experiments.

Within a large immunophenotyping screening program designed to detect genes involved in immune homeostasis, loss of the *Pepd* gene (*Pepd*^−/−^) by targeted mutagenesis was shown to be associated with the generation of autoantibodies (12). Initial screening of male and female *Pepd*^−/−^ mice demonstrated increased incidence of ANAs compared with WT controls at 12 wk (WT 67/386, 17.36% positive versus *Pepd*^−/−^ 6/14, 42.9% positive, *p* = 0.0267, two-tailed Fisher’s exact test), and a similar trend toward ANA positivity was recapitulated on rederivation of the mice in a separate facility (Fig. 1A; WT 3/38, 7.9% positive versus *Pepd*^−/−^ 9/40, 22.5% positive, *p* = 0.0739, two-tailed Fisher’s exact test), mirroring the rate of ANAs seen in humans with PD (21%) (7). SLE has a 9:1 female-to-male incidence (20). Likewise, we found a higher frequency of positive ANAs in female versus male *Pepd*^−/−^, both in the initial screening (4/7, 57.1% *Pepd*^−/−^ females versus 2/7, 28.6% *Pepd*^−/−^ males) and in the rederived colony (7/26, 26.9% *Pepd*^−/−^ females versus 2/14, 14.3% *Pepd*^−/−^ males) (Fig. 1A). The presence of a lupus band in the skin and specific ANA to dsDNA are also pathogenic features of SLE in patients and mouse models (21, 22); however, both were equivalently low in WT and *Pepd*^−/−^ mice (Supplemental Fig. 1A, 1B). C57BL/6 mice are known to be relatively resistant to renal injury (23, 24), and consistent with this, there was no evidence of impaired renal clearance as measured by serum creatinine (Supplemental Fig. 1C).

As previously reported, *Pepd*^−/−^ mice were smaller than their WT counterparts (Fig. 1B) (25). Murine *Pepd*^−/−^ serum IgA, but not IgG or IgM, was increased, with IgA, IgG, and C1q deposited in the renal glomeruli, a pattern consistent with the immune complex–mediated disease seen in human SLE (Fig. 1C–E).

There were lower numbers of *Pepd*^−/−^ cells in BM fractions A and E; otherwise, the numbers of conventional B2 B cell subsets in the BM and periphery of WT and *Pepd*^−/−^ mice were equivalent (Supplemental Fig. 1D–G). Despite the increased IgA serum titers and associated renal deposition, there were no significant differences in the numbers (Supplemental Fig. 1H, 1I) or proportions (data not shown) of IgA^+^ B cells or IgA^+^ plasma B cells in the spleen, mesenteric lymph node, or Peyer’s patches. Consistent with the initial Infection, Immunity and Immunophenotyping consortium phenotyping, we observed normal myeloid populations in the *Pepd*^−/−^ mice (12) (data not shown).

### PD results in expansion of activated peripheral T cells

**FIGURE 2.**
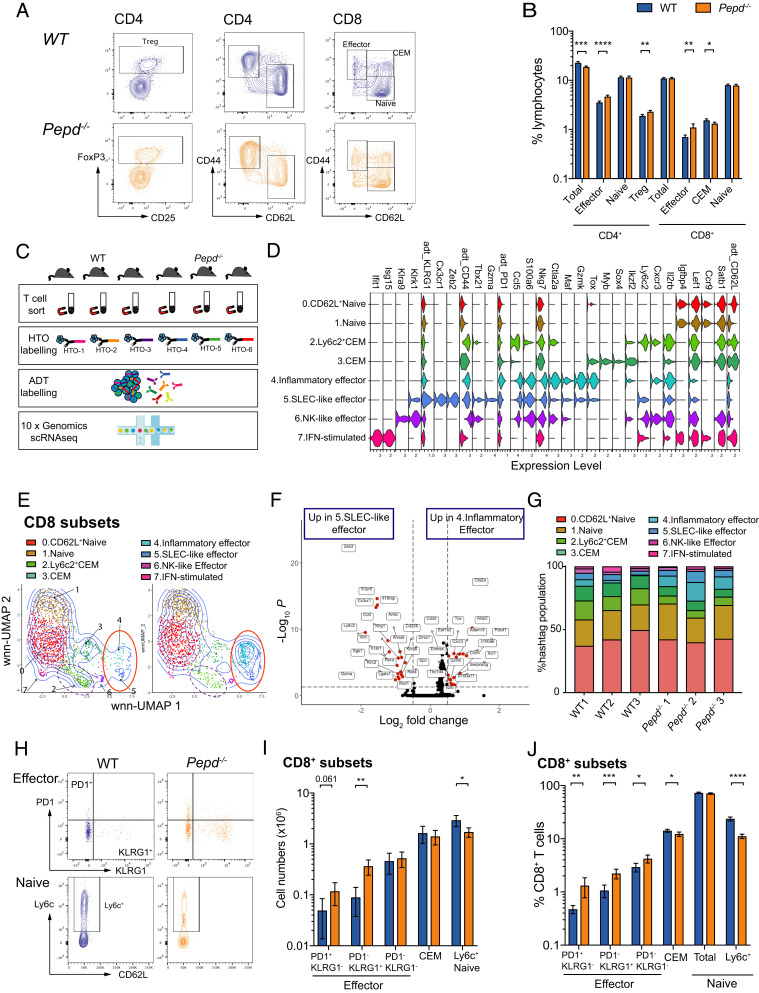
Prolidase deficiency results in cell-intrinsic expansion of activated peripheral T cells. (**A**) Representative flow cytometry plots of splenic CD4 and CD8 T cell subsets of WT and *Pepd*^−/−^ mice, previously gated on live, singlet cells. CEM, central effector memory CD8 cell; Treg, CD4 T regulatory T cell. (**B**) Pooled quantification of T cell subsets across 10–13 experiments each with 3–10 mice per group gated in (A) as proportion of splenic lymphocytes. Significance was determined as follows: total CD4, ***p = 0.0001; CD4 effector, ****p ≤ 0.00001; CD4 Treg, **p = 0.0027; CD8 effector, **p = 0.0027; CD8 CEM, *p = 0.0254. (**C**) Schematic of experiment. Splenic T cells from three WT and three *Pepd*^−/−^ mice were sorted using negative enrichment, then labeled with hashtag Abs, pooled, and labeled with 15 ADT T cell markers before 3′ single-cell transcriptomic sequencing. (**D**) Violin plot showing log expression of key RNA and protein (indicated by adt_) markers in WT and *Pepd*^−/−^ CD8 clusters defined by WNN clustering analysis. (**E**) Visualization of WT and *Pepd*^−/−^ CD8 populations described in (D) by WNN-UMAP dimensionality reduction and clustering, with populations of effectors ringed in red and Ly6c^+^ CEM populations in dashed purple. (**F**) Volcano plot depicting differential gene expression between inflammatory effectors (cluster 4) and SLEC-like effectors (cluster 5) of WNN-clustered CD8 populations from (D) and (E). Differentially expressed genes were determined using the Wilcoxon rank sum test and are colored in red at a threshold of log_2_FC = 0.5 and adjusted *p* value <0.05. (**G**) Quantification of the relative contribution of clusters identified in (D) and (E) as percentage of WT or *Pepd*^−/−^ total CD8 population by mouse. Data for (C)–(G) are from one scRNA-seq experiment. (**H**) Representative flow cytometry plots showing gating of CD8 splenic T cell populations in WT and *Pepd*^−/−^ mice. (**I**) Representative T cell numbers for WT and *Pepd*^−/−^ splenic CD8 populations gated as in (H); significance is depicted for representative data as determined by an unpaired two-tailed *t* tests, adjusting for multiple comparisons. PD1^+^KLRG1^−^ effector, *p* = 0.061; PD1^−^KLRG1^+^ effector, **p = 0.00198; Ly6C^+^ naive, *p = 0.0135. Data in (H) and (I) are representative of 7–13 independent experiments with between 2 and 10 mice per group for each experiment. Significance was determined between subsets across independent experiments using two-way ANOVA. PD1^+^KLRG1^−^ effector, *p* = 0.0020; PD1^−^KLRG1^+^ effector, *p* ≤ 0.0001. However, an interaction between experiment date and genotype (p < 0.0034) was detected; Ly6C^+^ naive, *p* ≤ 0.0001. (**J**) Quantification of splenic populations as a percentage of CD8 T cells in WT and *Pepd*^−/−^ mice gated as in (H), pooled from 7–13 independent experiments with 2–10 mice per group aged between 8 and 40 wk. Significance between groups was determined by an unpaired two-tailed *t* test adjusted for multiple testing. PD1^+^KLRG1^−^ effector, **p = 0.0081; PD1^−^KLRG1^+^ effector, ***p = 0.0002; PD1^−^KLRG1^−^ effector, *p = 0.0133; CEM, *p = 0.0245; Ly6C^+^ naive, ****p < 0.00001.

In contrast to the findings in B cells, flow cytometric analyses revealed an expansion of activated peripheral CD4 and CD8 T cells in the spleens of *Pepd*^−/−^ mice. Across 13 replicate experiments, *Pepd*^−/−^ mice showed increased numbers of CD8 effector T cells compared with WT and reduced numbers of total and naive CD8 T cells (two-way ANOVA; total CD8, *p* = 0.015; CD8 effector, *p* = 0.028; CD8 naive, *p* = 0.0082) (Fig. 2A). The percentages of splenic CD4 effector and T regulatory cell (Treg) populations were also increased in *Pepd*^−/−^ mice (Fig. 2A, 2B). Within the thymus, we saw reduced numbers of CD4^−^CD8^−^ double-negative fraction 4 (DN4) and CD4^+^CD8^+^ double-positive (DP) thymocytes (Supplemental Fig. 1J–L).

### Multimodal sequencing identifies both terminally differentiated and exhausted CD8 effector phenotypes, increased by prolidase loss

To characterize the intrinsically altered T cell populations more comprehensively, we performed 3′ scRNA-seq on HTO-multiplexed WT and *Pepd*^−/−^ T cells (Fig. 2C). Oligo-conjugated ADTs for 13 T cell markers were applied to strengthen the identification of subsets. In total, 14,568 cells were captured and sequenced, with postfiltering and demultiplexing steps leaving 8,220 singlets across the six samples, with an average of 1,783 genes detected per cell (Supplemental Fig. 2A, 2B). By subsetting and subsequent WNN clustering analysis using both ADT and RNA information (18), seven CD4 and eight CD8 populations were identified in both WT and *Pepd*^−/−^ mice, distinguished by naive, activation, localization, and memory markers (Fig. 2D, 2E, Supplemental Fig. 2C, 2D). Three CD4 populations had effector features, including high CD44 and low CD62L at the protein level (clusters 1, 3, and 6; Supplemental Fig. 2C, 2D).

Within WT and *Pepd*^−/−^ CD8 populations, scRNA-seq identified two transcriptionally distinct central effector memory (CEM) populations: *Ly6c2*^+^ CEM cells in cluster 2 could be characterized by higher expression of *Ly6c2*, *Il2rb*, and *Cxcr3* as well as effector molecules *Ccl5* and *Ctla2a*, whereas *Myb*, *Ikzf2*, and *Sox4* were highly expressed within CEM cluster 3 (Fig. 2D, Supplemental Fig. 2F).

Three effector CD8 T cell clusters, that is, clusters 4, 5, and 6, were identified by WNN clustering of both WT and *Pepd*^−/−^ populations (Fig. 2D, 2E). Cluster 6 was a small population characterized by expression of NK-like markers including *Klrk1* and *Klra9*. The larger clusters 4 and 5 were compared to identify distinguishing expression profiles. On this basis, cells in cluster 4 were defined as inflammatory effectors based on relatively higher expression of inflammatory transcripts, including *Gzmk*, *Cxcr3*, *Xcl1*, and S100a6, and exhaustion markers, such as *Ctla2a*, *Tox*, *Pdcd1*, and *Eomes* (Fig. 2D, 2F). Effector cells in cluster 5 were characterized by RNA expression of genes downstream of transcription factor *Tbet*, including *Zeb2*, *Cx3cr1*, and *S1pr5*, with high KLRG1 protein and RNA expression (Fig. 2D, 2F). This signature is comparable to the profile of terminally differentiated, short-lived effector cells (SLECs) observed during the early stages of viral infection or inflammation (26–31).

Consistent with previous flow cytometry experiments (Fig. 2A, 2B), quantitative population analysis between WT and *Pepd*^−/−^ CD8 clusters identified significant reductions in both CEM populations in *Pepd*^−/−^ mice, whereas there was a trend toward increased representation of *Pepd*^−/−^ cells within CD8 inflammatory effector populations (Fig. 2G). Markers identified by scRNA-seq were then applied to confirm these findings at the protein level, KLRG1 SLEC-like effectors, and PD1 as a marker of both chronic inflammation and potential exhaustion (32) (Fig. 2H). Flow cytometry demonstrated that numbers and proportions of both PD1^+^ and KLRG1^+^ splenic effectors were increased in *Pepd*^−/−^ mice (Fig. 2I, 2J). Additionally, transcriptomic analysis demonstrated significantly reduced Ly6C^+^ CEM and CEM populations within *Pepd*^−/−^ CD8 T cells (Fig. 2G), and similarly staining for Ly6C by flow cytometry highlighted a significant reduction in *Pepd*^−/−^ Ly6C-expressing CD62L^+^ populations as numbers and proportions of CD8 cells (Fig. 2H–J).

Differential expression analysis between WT and *Pepd*^−/−^ cells demonstrated that alongside *Pepd*, *Dapl1* and *Ifi27l2a* were significantly downregulated in *Pepd*^−/−^ CD4 and CD8 naive cells (Supplemental Table I). *Dapl1* has been implicated as a critical regulator of CD8 T cell activation and exhaustion (33), whereas *Ifi27l2a* is associated with increased susceptibility to bacterial infections and specific viral infections (34, 35). In *Pepd*^−/−^ inflammatory effector cells, alongside increased expression of inflammatory and effector molecules *S100a4*, *S100a6*, *Lgals1*, *Arap2*, and *Ahnak* (36–38), the survival marker *Bcl2* was significantly upregulated (Supplemental Table I). *Ly6c2* was also significantly reduced across multiple *Pepd*^−/−^ CD4 and CD8 T cell clusters compared with WT (Supplemental Table I).

### Cell-intrinsic prolidase loss drives the activated T cell phenotype

**FIGURE 3.**
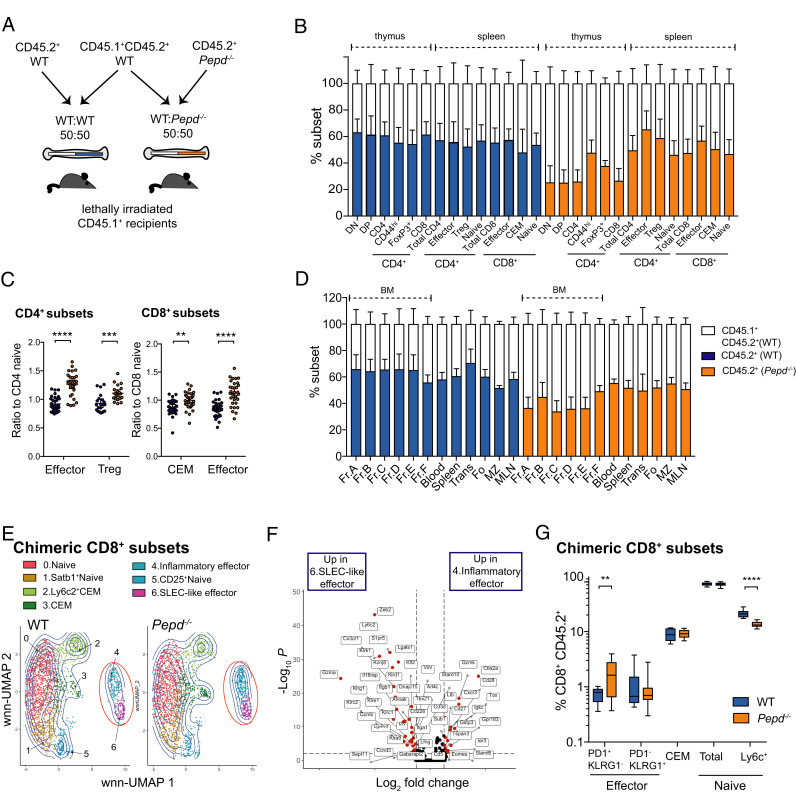
Effector CD8 T cells with an inflammatory, exhausted profile are associated with intrinsic prolidase deficiency. (**A**) Schematic of bone marrow chimera experiment. CD45.1^+^ recipients were lethally irradiated and reconstituted for 8 wk with equal amounts of CD45.1^+^CD45.2^+^ WT and either CD45.2^+^ WT or CD45.2^+^ *Pepd*^−/−^ BM. (**B**) Relative proportions of thymic populations and splenic T cell subsets from BM chimeras reconstituted with equal ratios of CD45.1^+^CD45.2^+^ WT and either CD45.2^+^ WT or CD45.2^+^ *Pepd*^−/−^ BM, end-gated based on CD45.1 and CD45.2. Data show one experiment representative of four independent repeats. (**C**) Ratios of proportions of specified CD4 or CD8 cell populations to naive subset within WT or *Pepd*^−/−^ CD45.2^+^ splenic CD4 and CD8 T cells from chimeric reconstituted BM, pooled from six experimental replicates. CD4 effector, ****p ≤ 0.0001; CD4 Treg, ***p = 0.00031; CD8 CEM, **p = 0.00131; CD8 effector, ****p ≤ 0.00001. (**D**) Relative proportions of B cell reconstitution across organs in BM chimeras described in (A), representative of three independent repeats. (**E**) WT and *Pepd*^−/−^ CD45.2^+^ T cells from BM chimeras described in (A) were sorted using flow cytometry and negative enrichment, then labeled with hashtag Abs, pooled, and labeled with 13 ADT T cell markers before 3′ single-cell transcriptomic sequencing. WT and *Pepd*^−/−^ CD8 populations were identified by WNN-UMAP dimensionality reduction and clustering, with populations of effectors ringed in red. (**F**) Volcano plot depicting differential gene expression between inflammatory effectors (cluster 4) and SLEC-like effectors (cluster 6) of WNN-clustered CD8 populations from (E). Differentially expressed genes were determined using the Wilcoxon rank sum test and are colored in red at a threshold of log_2_FC = 0.5 and adjusted *p* value <0.01. Data for (E) and (F) are from one scRNA-seq experiment. (**G**) Flow cytometric determination of proportions of WT and *Pepd*^−/−^ splenic CD45.2^+^ CD8 subsets of chimeras reconstituted as in (A), pooled from three independent experiments, each with five to six mice per group and significance determined by an unpaired two-tailed *t* test, adjusting for multiple comparisons. PD1^+^KLRG1^−^ effector, **p = 0.0055; Ly6c^+^ naive, ****p ≤ 0.0001.

Because prolidase is ubiquitously expressed, it was important to confirm whether the T cell immunophenotype was intrinsic or due to external effects of prolidase loss. To assess this we reconstituted lethally irradiated, allotype-marked CD45.1^+^ recipients with 50:50 mixtures of double-labeled CD45.1^+^CD45.2^+^ WT and either CD45.2^+^*Pepd*^−/−^ or CD45.2^+^ WT control BM (Fig. 3A). *Pepd*^−/−^ BM was reproducibly less effective than WT BM in the reconstitution of thymic T cells and BM B cells (Fig. 3B, 3D), possibly due to a relative defect in hematopoietic stem cell function, consistent with some observations in unmanipulated mice (Supplemental Fig. 1E, 1K). Despite this, in the periphery, we observed a pattern of higher *Pepd*^−/−^ contribution to activated T cell subsets in the mixed chimeras, with expansion of CD45.2^+^ CD4 effector cells, Tregs, and CD8 effector and CEM populations, relative to naive *Pepd*^−/−^ CD4 or CD8 subset proportions, respectively (Fig. 3B, 3C). In contrast, there was no difference in the ability of *Pepd*^−/−^ and WT B cells to populate the periphery (Fig. 3D).

Despite the evidence for an intrinsic effector T cell phenotype, serum IgA titers were not significantly elevated in WT:*Pepd*^−/−^ chimeras (WT:WT, 331.3 ± 35.77 μg/ml, *n* = 16; WT:*Pepd*^−/−^, 598.9 ± 250.1 μg/ml CEM, *n* = 16, *p* = 0.2980, unpaired *t* test), ANAs were not elevated in the serum of chimeric mice (WT:WT, 0/16 positive; WT:*Pepd*^−/−^, 1/16 positive; Fisher’s exact test, *p* = 0.99), and we did not find increased IgA deposition in the kidneys of chimeric mice (data not shown).

The transcriptional profiles identified in WT and *Pepd*^−/−^ effector CD8 T cells were mirrored in chimeric WT and *Pepd*^−/−^ CD45.2^+^ populations in a secondary scRNA-seq experiment (Fig. 3E, 3F, Supplemental Fig. 3A), including PD1^+^ inflammatory and KLRG1^+^ SLEC-like effectors, indicating that these profiles are robust across chimeric and nonchimeric mice. However, although flow cytometry analysis demonstrated that Ly6C^+^ naive populations were comparably reduced within chimeric CD45.2^+^ *Pepd*^−/−^ CD8 cells, inflammatory effectors were the only effector population enriched in chimeric CD8 CD45.2^+^ *Pepd*^−/−^ populations, whereas proportions of SLEC-like effectors were equivalent, as were CEM cell proportions (Fig. 3G). There were no differences in CD45.1^+^CD45.2^+^ WT populations from WT:WT or WT:*Pepd*^−/−^ chimeras (Supplemental Fig. 3B), justifying sequencing of CD45.2^+^ populations only.

Despite effector and naive cell alterations at the population level between chimeric WT and *Pepd*^−/−^ CD8 T cells, a small number of genes were differentially expressed between WT and *Pepd*^−/−^ CD8 T cells in both chimeric and nonchimeric experiments. *Pepd* and *Ly6c2* were consistently lower within naive chimeric (*Pepd*, −3.54 log fold change [FC], *p* = 7.94E−16; *Ly6c2*, −1.03 logFC, *p* = 1.14E−10) and nonchimeric *Pepd*^−/−^ CD8 cells (Supplemental Table I), and *Klre1* was reduced in chimeric (−1.28 logFC, *p* = 2.44E−05), and nonchimeric *Pepd*^−/−^ Ly6C^+^ CEM populations (Supplemental Table I).

### Increased differentiation into CD8 T cell effectors in PD

Our findings indicate that PD causes an accumulation of CD8 effector T cells with an inflammatory signature in a cell-intrinsic manner, rather than due to extrinsic effects from other cell types, such as altered Ag processing and presentation by other cells. This is distinct from the accumulation of KLRG1^+^ SLEC-like effectors, which is rescued in the presence of WT cells. Expansion of CD8 effector populations could be explained by a number of possibilities. Loss of prolidase could extend the survival of effector populations, or enhance their intrinsic ability to differentiate or proliferate, spontaneously or in response to self-antigens or foreign Ags.

**FIGURE 4.**
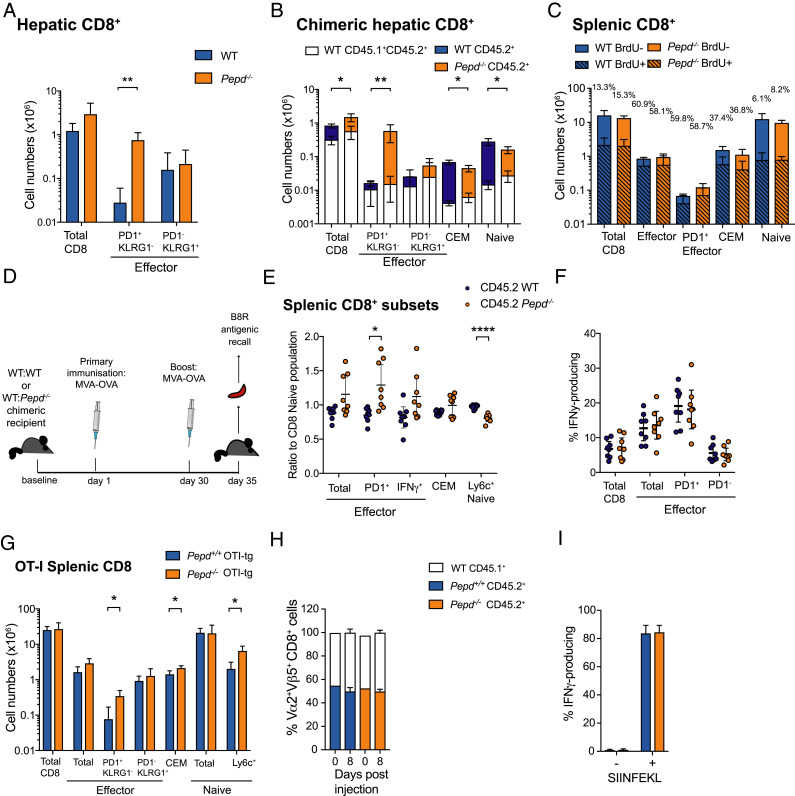
*Pepd*^−/−^ effectors accumulate in sites of likely breakdown due to enhanced Ag-independent proliferation. (**A**) Representative numbers of CD8 cells in the livers of WT or *Pepd*^−/−^ mice, with significance for representative data determined by an unpaired two-tailed *t* test adjusted for multiple comparisons. PD1^+^KLRG1^−^ effectors, **p = 0.00235. Data are representative of three independent experiments, each performed with three to four mice per group sex and age matched between 10 and 17 wk. Significance between groups for cell subsets was determined by a two-way ANOVA across independent experiments. Total CD8, *p* = 0.0299; PD1^+^KLRG1^−^ effector, *p* ≤ 0.00001; an interaction between experimental date and genotype (*p* < 0.0001) was detected for PD1^+^KLRG1^−^ effector populations. (**B**) Mixed BM chimeras reconstituted with congenically labeled WT:WT BM or WT:*Pepd*^−/−^ BM. Representative quantification is shown of absolute numbers of WT CD45.1^+^CD45.2^+^ (white) and WT CD45.2^+^ (blue) or *Pepd*^−/−^ CD45.2^+^ (orange) hepatic CD8 populations. Significance for representative data was determined by an unpaired two-tailed *t* tests adjusted for multiple comparisons for total chimeric cells (CD45.1^+^CD45.2^+^ and CD45.2^+^). CD8, *p = 0.03; PD1^+^KLRG1^−^, **p = 0.0057; CEM, *p = 0.0156; Ly6c^+^ naive, *p = 0.0147. Bars indicate means, and error bars represent 95% confidence intervals (CIs). Data are representative of three independent experiments with three to six mice per group, with significance determined for subsets of total chimeric cells by two-way ANOVA across independent experiments. PD1^+^KLRG1^−^ effector, *p* = 0.0341. (**C**) Numbers of BrdU-labeled and unlabeled WT or *Pepd*^−/−^ CD8 cells. Data are representative of two experiments with three to four mice per group, sex and age matched between 10 and 24 wk. (**D**) Schematic of immunization program for mixed WT:WT or WT:*Pepd*^−/−^ BM chimeric mice with MVA-OVA. B8R is an immunodominant MVA-derived epitope. (**E**) CD45.2^+^ WT and *Pepd*^−/−^ CD8 subsets as ratios to CD8 CD45.2^+^ naive proportions in the spleen of mixed BM chimeras immunized as in (D), pooled from two experiments with four mice per group. Significance was determined by an unpaired two-tailed *t* test, adjusting for multiple testing. PD1^+^effector, *p = 0.0206; Ly6c^+^ naive, ****p ≤ 0.00001. (**F**) Functional IFN-γ production on B8R peptide-stimulated recall of WT or *Pepd*^−/−^ CD45.2^+^ CD8 splenocyte populations isolated from chimeras immunized as in (D), measured by intracellular cytokine staining, pooled from two independent experiments with four mice per group. For (E) and (F), symbols represent individual mice with 95% CI error bars. (**G**) Representative absolute numbers of CD8 T cell subsets from WT and *Pepd*^−/−^ OT-I tg mice with significance for representative data determined by an unpaired *t* test adjusted for multiple testing. PD1^+^KLRG1^−^ effector, *p = 0.020; CEM, *p = 0.028; Ly6c^+^ naive, *p* = 0.0147. Bars are means with 95% CI error bars; data are representative of three independent experiments with three to four mice, sex and age matched between 9 and 20 wk. Significance was determined by a two-way ANOVA across these experiments. Total effector, *p* = 0.0146; PD1^+^KLRG1^−^ effector, *p* = 0.0054; CEM, *p* = 0.0242; Ly6c^+^ naive, *p* = 0.0012. (**H**) Ratios (50:50) of WT:WT or WT:*Pepd*^−/−^ OT-I tg cells were injected into recipient mice, then immunized with MVA-OVA. Resulting ratios of WT:WT or WT:*Pepd*^−/−^ within Vα2^+^Vβ5^+^ CD8 T cells from mice culled on day 8 were determined by flow cytometry. (**I**) Intracellular IFN-γ production on recall of WT or *Pepd*^−/−^ splenocytes isolated from (H) with SIINFEKL peptide. For (H) and (I), data are representative of three independent experiments with four to five mice per group. Graphical data show mean at bar and 95% CI error bars.

Activated lymphocytes are removed at many important sites of peripheral tolerance. In particular, the liver represents a key immunological organ that acts to prevent autoimmunity, partly by silencing and removing T cells by inducing Bim-mediated apoptosis (39–42). Flow cytometric analysis of lymphocyte populations in *Pepd*^−/−^ liver identified increased numbers of CD8 PD1^+^ effectors relative to WT (Fig. 4A). The causes of this accumulation in the liver are cell intrinsic because absolute numbers of PD1^+^ effectors were significantly increased in the livers of mixed WT:*Pepd*^−/−^ BM chimeric recipients compared with WT:WT recipients (Fig. 4B). Importantly, however, the relative accumulation of cells was similar in the liver and in the spleen (Fig. 2B). Despite increased Bcl2 RNA expression in *Pepd*^−/−^ PD1^+^ effectors (Supplemental Table I), this was not observed at the protein level (data not shown), and no transcriptional differences in survival pathways were detected in chimeric populations between WT and *Pepd*^−/−^ CD8 splenic effector T cells. Protein expression of the survival marker Bcl2, and apoptotic protein Bim, were also equivalent across hepatic and splenic WT and *Pepd*^−/−^ CD45.2^+^ CD8 chimeric populations (Supplemental Fig. 3C–F).

The equivalent accumulation of *Pepd*^−/−^ CD8 T cell populations in the liver compared with spleen, and lack of consistent, observable differences in survival pathways between WT and *Pepd*^−/−^ T cell populations, suggested that the increase in CD8 PD1^+^ T cell effectors might be due to enhanced differentiation or proliferation in the absence of prolidase rather than reduced cell loss. We sought to investigate this by administering BrdU orally for 1 wk to WT or *Pepd*^−/−^ mice. Although the *Pepd*^−/−^ CD8 PD1^+^ effector T cell population was expanded as before, the percentage of BrdU-labeled cells was the same as in WT (Fig. 4C). Cell labeling with the proliferation marker Ki-67 also showed a similar percentage of CD8 PD1^+^ effector cells in WT and *Pepd*^−/−^ unmanipulated and chimeric mice (Supplemental Fig. 3G, 3H). Taken together, these findings indicate an equivalent proliferation rate within the effector population. In the absence of evidence for altered survival, these observations suggest that expansion of the PD1^+^ effector pool is most likely due to increased differentiation into the subset. We conclude that PD results in an intrinsic increase in the number of cells adopting an inflammatory effector CD8 T cell phenotype.

### The accumulation of *Pepd*-deficient CD8 effector T cells is Ag-independent

To explore the possible role of Ag in driving increased numbers of prolidase-deficient CD8 effector populations, 50:50 WT:WT and WT:*Pepd*^−/−^ mixed chimeras were immunized and boosted after 30 d with MVA encoding OVA (MVA-OVA) (Fig. 4D). The baseline *Pepd*^−/−^ CD8 T effector phenotype was unchanged in immunized WT:*Pepd*^−/−^ BM recipients (Fig. 4E), and the proportions of *Pepd*^−/−^ CD8 populations that produced IFN-γ were equivalent in CD45.2^+^ WT and *Pepd*^−/−^ cells (Fig. 4F), indicating that there was no intrinsic effect of PD on the functional response to a T_H_1-associated foreign Ag.

The role of TCR expression and specificity in driving T cell subset differentiation was investigated by crossing *Pepd*^−/−^ mice to the OT-I strain where CD8 T cells express a TCR transgene (tg) specific for the MHC class I SIINFEKL epitope of OVA (43). Splenic *Pepd*^−/−^ PD1^+^ effector CD8 T cells still accumulated in the presence of the OT-I tg, consistent with this phenomenon being independent of TCR specificity or exposure to Ag (Fig. 4G). However, the KLRG1^+^ effector population and the splenic *Pepd*^−/−^ Ly6C^+^ naive population, observed to be significantly altered in nontrangenic *Pepd*^−/−^ mice (Fig. 3F), were both rescued in the OT-I *Pepd*^−/−^ model (Fig. 4G). Although the nontransgenic chimeric data suggest that KLRG1^+^ CD8 expansion may also require cell-extrinsic consequences of PEPD loss, the rescue of the both the KLRG1^+^ and Ly6C phenotypes in a nonchimeric, TCR tg context may depend on TCR specificity or signal strength, and indicates that reduction in Ly6C^+^ naive T cells is not directly linked to the accumulation of PD1^+^ effectors.

To confirm the absence of a role for TCR signaling by self-peptide, we transferred 50:50 mixtures of OT-I tg WT and *Pepd*^−/−^ cells into nontransgenic recipients and immunized them with the cognate Ag MVA-OVA. Seven days after immunization, ratios of WT:WT and WT:*Pepd*^−/−^ OT-I Ag-specific cells were unaltered from baseline, suggesting no direct effect of prolidase on the response to self-antigen (Fig. 4H). As with foreign Ag in nontransgenic populations, *Pepd*-deficient MVA-OVA–immunized CD8 OT-I tg T cells showed equivalent IFN-γ production in response to stimulation with the SIINFEKL peptide ex vivo (Fig. 4I).

Collectively, these data suggest that intrinsic prolidase loss drives a nonspecific increase in the number of CD8 T cell effectors expressing inflammatory and exhaustion markers, independent of exposure to either self-antigen or foreign Ag. However, extrinsic and prolidase-dependent factors are required for regulating SLEC-like CD8 effector populations, alongside systemic hallmarks of autoimmunity.

## Discussion

The role of the metallopeptidase prolidase in regulating immune tolerance has previously been suggested by the variable autoimmune syndrome seen in some prolidase-deficient human patients. The incidence of ANAs in humans with PD is estimated at 21% (7); consistent with this, and with the expected sexual dimorphism, we observed positive ANAs in 42.9% of *Pepd*^−/−^ mice on initial screening, and 22.5% when these animals were rederived at our institution. Because WT ANA frequency was also lower in the second location, this likely to be due to environmental differences between the two animal facilities.

Prolidase-deficient mice also demonstrate an activated T cell phenotype and elevated serum IgA, with glomerular immune complex deposition reminiscent of early human lupus nephritis (44), mimicking the phenotype in a subset of PD patients (7, 8, 11, 45) and confirming, to our knowledge, for the first time, the direct association of autoimmunity and PD. However, loss of prolidase did not result in impaired renal function, nor did we detect immune complex deposition in other organs. The renal location of immune complex deposition may be due to some unknown renal specific self-antigen, or in the context of increased serum IgA, due to trapping of the preformed immune complex within the renal glomerulus (46, 47). Thus the phenotype is of autoimmunity without clinically significant end organ damage; C57BL/6 mice are known to be relatively resistant to renal injury (23, 24), and therefore we cannot exclude a more overt renal phenotype due to PD with a more susceptible background strain.

In the absence of prolidase, both CD4 and CD8 effector T cells accumulate, whereas peripheral B cell numbers are normal. Consistent with our findings in the *Pepd*^−/−^ mice, immune profiling of two prolidase-deficient patients has described normal B cell numbers, accompanied by an increase in CD8 effector T cells with an inflammatory phenotype in one of these patients (48).

Similar immunophenotyping of mice with PD identified that the most striking accumulation of cells occurred in the CD8 T cell compartment, and so this is where we focused our attention. In WT and *Pepd*^−/−^ mice, CD8 effectors have a transcriptional profile characteristic of either SLEC-like terminal differentiation or chronic inflammation including features of exhaustion. Comparable patterns of transcriptional exhaustion have been reported in models of other autoimmune diseases, including type I diabetes and nonalcoholic steatohepatitis (30, 42, 49). Both SLEC-like and inflammatory effector populations were significantly enlarged in unmanipulated *Pepd*-deficient mice, where Ly6C^+^ naive populations were reduced. Although Ly6C^+^ naive and PD1^+^ effector populations were demonstrated to be affected in an intrinsic manner, KLRG1^+^ SLEC-like effectors were not, and this population was rescued in mixed chimeric WT:*Pepd*^−/−^ mice alongside loss of hallmarks of autoimmunity.

Furthermore, the alterations to Ly6C^+^ naive cells and KLRG1^+^ effectors were also rescued in *Pepd*^−/−^ OT-II^+^ mice, indicating a role for TCR specificity in these immunophenotypes. However, the PD1^+^ effector phenotype was unchanged by expression of the OVA-specific TCR tg or immunization, and WT and prolidase-deficient mice responded equivalently to self-Ag and foreign Ag despite these inflammatory effector populations. The driver of the inflammatory effector phenotype remains unclear; although the number of PD1^+^ CD8 effector T cells did increase in the liver, the expansion was similar to that seen elsewhere in the periphery, and there was no evidence for altered apoptosis or survival in these populations, suggesting no intrinsic or extrinsic defect in cell clearance. However, we cannot fully exclude defective clearance by a non–Bim/Bcl2-mediated cell-intrinsic pathway, such as invasion of hepatocytes by autoreactive T cells leading to endosomal degradation (39). The percentage of divided cells was identical in splenic *Pepd*^−/−^ and WT effector CD8 T cell populations, suggesting that increased differentiation of cells into the effector phenotype is the most likely explanation for expansion of these populations in the absence of prolidase.

The nature of the intrinsic T cell defect may have its explanation in previously described downstream effects of prolidase enzymatic action, such as the stabilization of HIF-1α (50). Independent of iminodipeptide metabolism, prolidase may regulate p53 availability and therefore influence apoptosis (51), and act as an extracellular ligand of ErbB2 to inhibit Src signaling and tumorigenesis (51–53). However, no transcriptomic evidence for altered HIF-1α, p53, or Src signaling was detected within *Pepd*^−/−^ T cells, and cell survival signals were equivalent, suggesting that novel posttranslational mechanisms may be driving the altered T cell fates detected in these experiments.

A specific population of KLRG1^+^ CD8 effectors was only increased in the nonchimeric mice, indicating dependence on cell-extrinsic effects for both this population accumulation, as well as the associated pathological phenotype of ANAs, elevated serum IgA, and renal IgA immune complex deposition. Although CD4 and CD8 effector T cells with an inflammatory phenotype were expanded in the WT:*Pepd*^−/−^ chimeric mice, the proportion of SLEC-like effector cells and serum IgA and ANA production were normal, highlighting a potential relationship between disease activity of SLEC-like effectors and hallmarks of autoimmunity in the absence of prolidase. The development of autoimmunity may therefore additionally require cell-extrinsic consequences of PD, which could include the expansion of SLEC-like effectors or absence of Tregs, as WT Tregs will be present in mixed chimeras. The development of a T cell–specific *Pepd* knockout model would help to address this question. As autoimmunity is not fully penetrant in human PD patients, and it presents as a biochemical and histological, rather than clinical, phenotype in prolidase-deficient mice, it is possible that loss of prolidase provides a first hit on lowering the threshold for autoimmunity, but a second challenge is required for loss of self-tolerance. The high rate of chronic skin and respiratory infections may provide this second hit in some patients.

PD is a very rare, albeit likely underdiagnosed, disorder, yet its association with SLE suggests that the elucidation of prolidase function may hold important clues for understanding more complex forms of autoimmune disease. Our findings and the *Pepd*^−/−^ model present novel insights and a new entry point for studying autoimmunity and tolerance. The signatures of chronic inflammation and exhaustion shed new light on T cell effector function, and they identify a targetable pathway, which may be relevant to both PD and complex autoimmune disease.

## Supplementary Material

Supplemental 1 (PDF)

Supplemental 1 (XLSX)
