# Supplementary material for: Prolidase Deficiency Causes Spontaneous T Cell Activation and Lupus-like Autoimmunity
Source: J Immunol. 2023 Jan 11;210(5):547–57. doi: 10.4049/jimmunol.2200212 (PMC9946897; doi:10.4049/jimmunol.2200212)
Supplement: Supplemental 1 (PDF) [file JI_2200212_Supplemental_1.pdf]

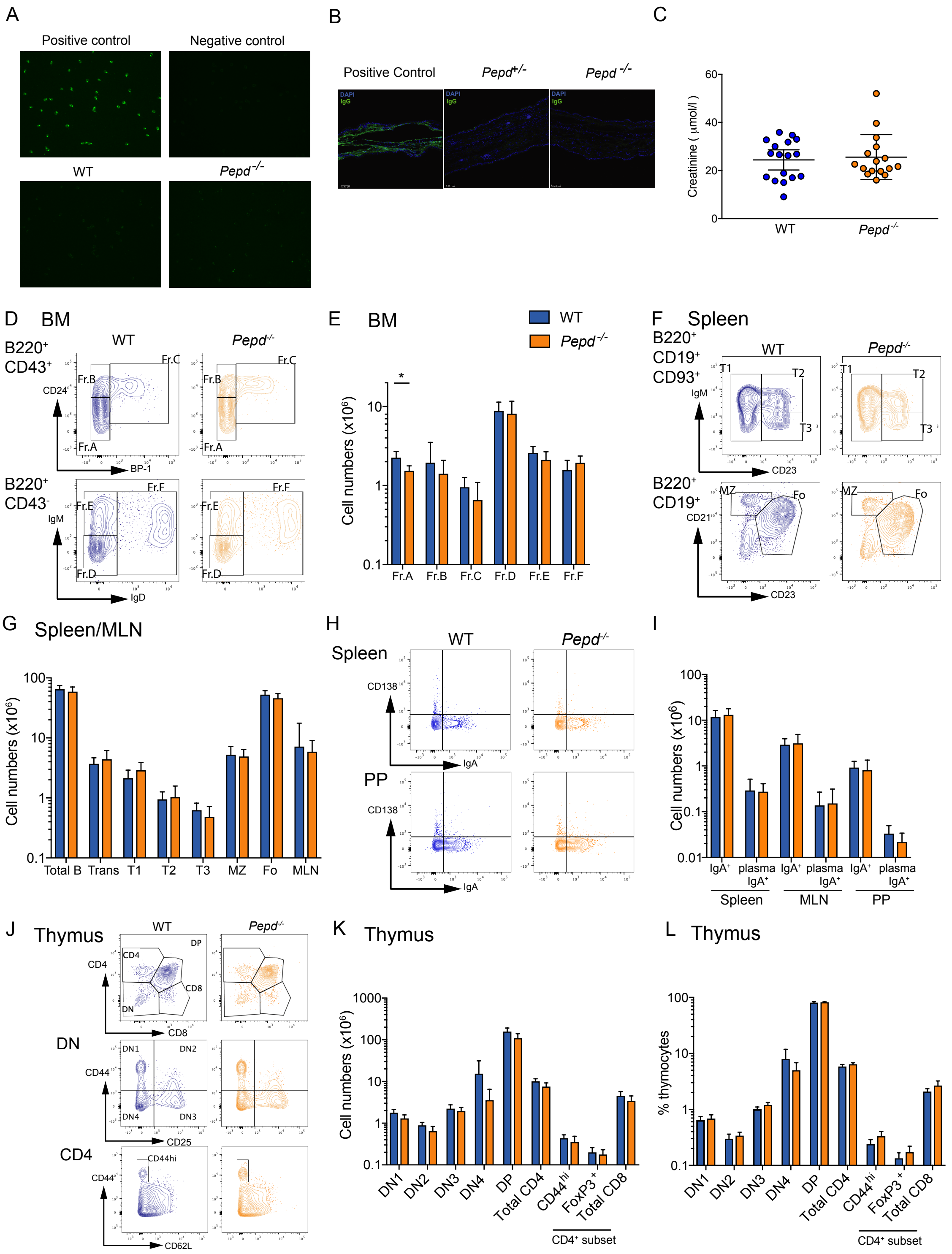

Supplemental Figure 1. *Pepd*$^{-/-}$ mice show normal B and T cell development and differentiation

A. Representative images of dsDNA detection in serum from positive control (BM12), negative control (C57BL/6 mouse positive for ANA, negative for anti-dsDNA), WT and *Pepd*$^{-/-}$ mice, with positive staining identified in green. Data are from one dsDNA assay of 21 WT and 24 *Pepd*$^{-/-}$ mice collected from 4 independent experiments. B. Representative images of lupus band detection in the ear epidermis in positive control (MRL/Fas), *Pepd*$^{+/-}$ and *Pepd*$^{-/-}$ mice, with positive IgG staining (green) and DAPI counter-stain (blue). Data are from one experiment, 6 *Pepd*$^{-/-}$ mice and 3 *Pepd*$^{+/-}$ controls. C. Serum creatinine levels from WT and *Pepd*$^{-/-}$ mice. Each point is one mouse, assay was performed once on serum collected in 5 independent experiments. D. Representative flow cytometry plots of B cell development through Hardy fractions (Fr. A-F) in the BM of WT and *Pepd*$^{-/-}$ mice, populations were previously gated on live cells. E. Quantification of bone marrow (BM) B cell numbers as gated in (D), significance for representative data determined by unpaired two-tailed t-test adjusted for multiple testing; Fr.A * P=0.0262. Data representative of 3-4 independent experiments with 3-5 mice per group, significance was determined between subsets across independent experiments using two-way ANOVA; Fr. A P=<0.00001, Fr. E P=0.0054. F. Representative flow cytometry plots of splenic B cell populations of WT and *Pepd*$^{-/-}$ mice and mesenteric lymph node total B cells (MLN), quantified as representative cell numbers in (G). Abbreviations defined as marginal zone (MZ) cells, follicular B cells (Fo) and transitional B cell (trans, T1-3) subsets. H. Representative flow cytometry plots of IgA and CD138 staining on B220^{+} CD19^{+} gated cells across lymphoid organs including Peyer's patches (PP), quantified by gating of IgA^{+} B cells and plasma IgA^{+} B cells as representative absolute cell numbers in (I). Data in (F-I) are representative of 2-5 independent experiments, each with 3-6 mice per group. J. Representative flow cytometry plots of T cell development in the thymus of WT and *Pepd*$^{-/-}$ mice. Abbreviations defined as double negative (DN), double positive (DP). K. Representative quantification of thymus T cell numbers as gated in (J) with significance for representative data determined by unpaired t-test, adjusted for multiple testing. Total CD4 represents CD4^{+}CD8^{-} thymocytes and total CD8 represents CD4^{-}CD8^{+} thymocytes, as gated in (J, top panel). Two-way ANOVA was used for significance analysis of numbers (K) across experiments: DN4 P=0.0352, DP P=0.0152. L. Pooled quantification of populations gated as in (J) as proportion of thymocytes, unpaired two-tailed t-tests were used to determine significance across pooled subsets adjusting for multiple testing. Data in J-L is representative of 2-4 independent experiments, each with 3-5 mice per group. Columns represent means with 95% CI error bars. All controls were age and sex-matched within experiments, ranging from 8 to 27 weeks.

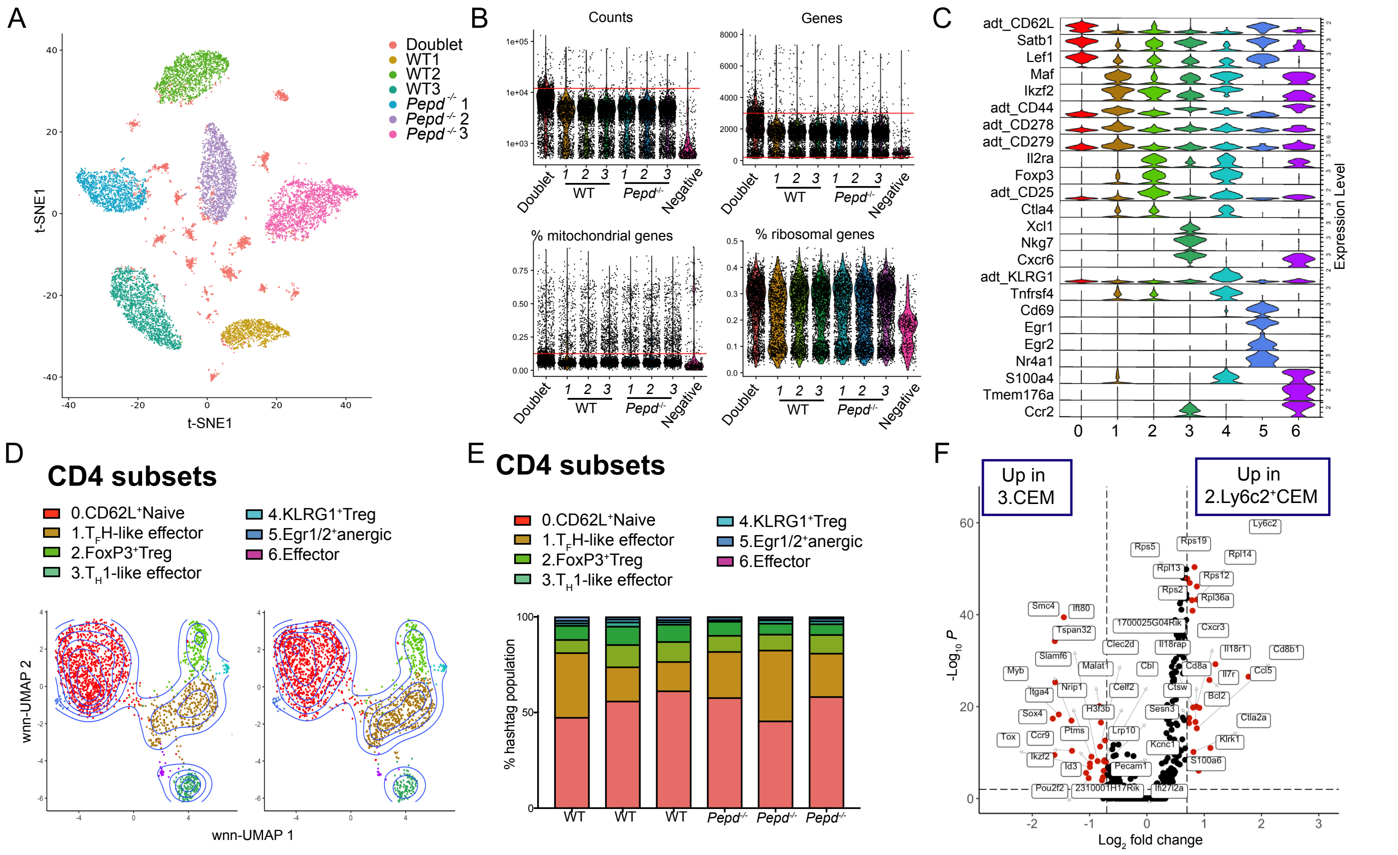

Supplemental Figure 2. Multimodal scRNAseq of WT and *Pepd-/-* T cells identifies transcriptional signatures of T cell effectors
A. WT or *Pepd-/-* samples were demultiplexed for downstream analysis based on hashtag expression, alongside heterogenous doublet removal. B. Singlets were filtered based on RNA (UMI) and gene (feature) counts per cell, and on mitochondrial gene expression. Thresholds shown as red lines, ribosomal gene expression is visualized for quality control. C. Violin plot of expression of RNA and protein markers in WT and *Pepd-/-* CD4 populations identified by WNN clustering. D. Visualisation of WNN-clustering analysis of WT and *Pepd-/-* CD4 populations in (C) by WNN-UMAP dimensionality reduction. E. Quantification of the relative contribution of clusters identified in (C and D) as percentage of each hashtagged CD4 sample population. F. Volcano plot depicting differential gene expression between Ly6c2+CEM cells (cluster 2) and CEM cells (cluster 3) of WNN-clustered CD8 populations from (Fig. 2D and E). Differentially expressed genes were determined using the Wilcoxon rank sum test and are coloured in red at a threshold of $log_2FC = 0.7$ and adjusted p value <0.01. Data are from one scRNAseq experiment.

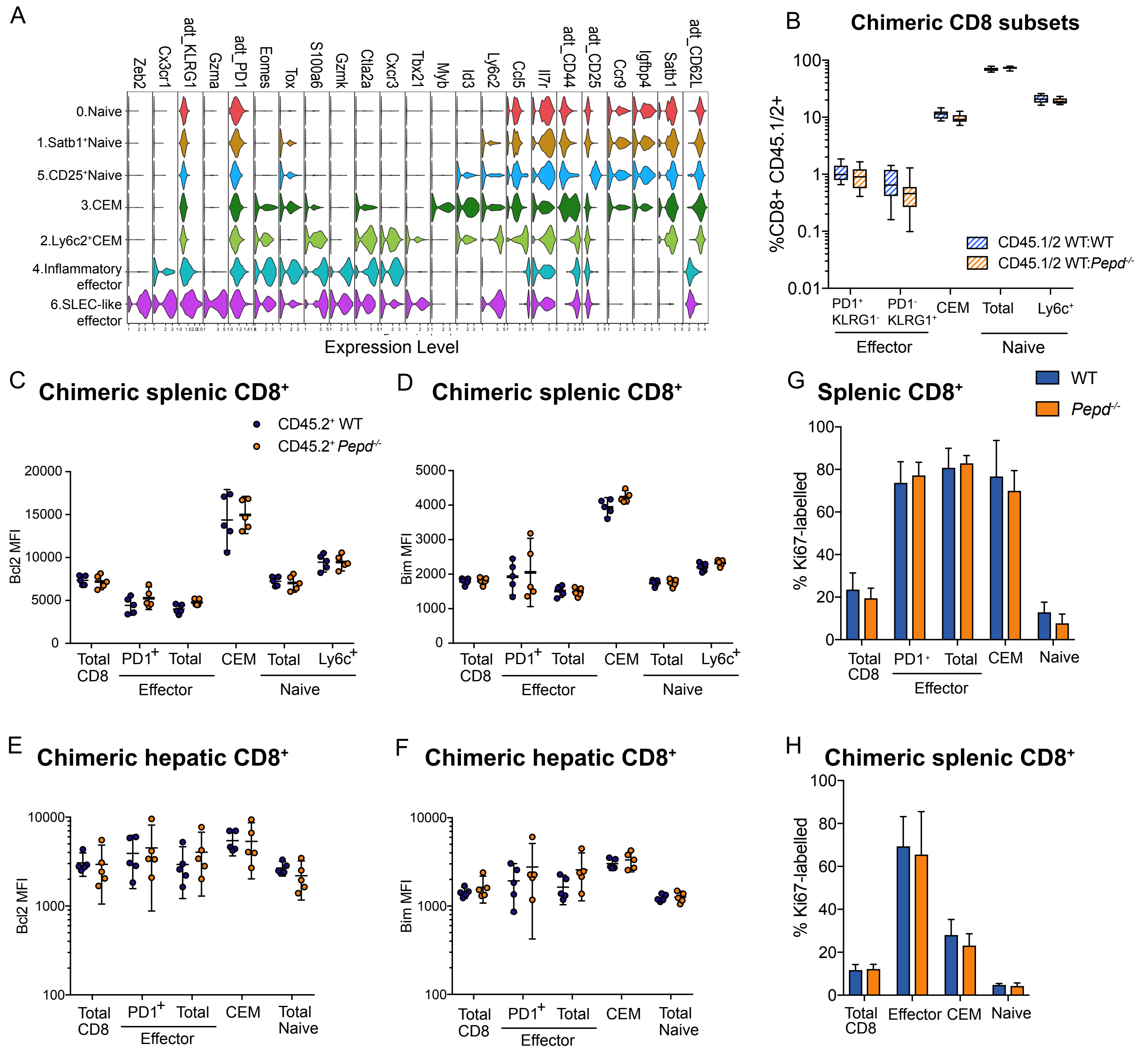

Supplemental Figure 3. Prolidase loss is associated with equivalent survival and apoptosis, but increased differentiation into the CD8 PD1^{+} effector pool

A. Violin plot of expression of RNA and protein markers in chimeric WT and *Pepd*$^{-/-}$ CD8 populations identified by WNN clustering from (Fig. 3E). Data are from one scRNAseq experiment. B. Flow cytometric determination of proportions of WT splenic CD45.1^{+} CD45.2^{+} CD8 subsets from WT:WT and WT:*Pepd*$^{-/-}$ chimeras reconstituted as in (Fig. 3A), pooled from three independent experiments, each with 5-6 mice per group and significance determined by unpaired two-tailed t-test, adjusting for multiple comparisons. C. Intracellular Bcl2 or D. Bim expression for chimeric splenic WT or *Pepd*$^{-/-}$ CD45.2^{+} CD8 populations. E. Bcl2 or F. Bim quantified as in (C and D) for hepatic WT or *Pepd*$^{-/-}$ CD45.2^{+} CD8 populations from chimeric mice. Data are representative for 2 independent experiments with 5-6 mice per group, points represent MFI of individual chimeric mice with 95% confidence intervals. Statistical significance was determined by unpaired two-tailed t-test, adjusting for multiple comparisons across subsets. G. Intranuclear staining of Ki-67 antigen in WT or *Pepd*$^{-/-}$ splenic CD8 subsets from non-chimeric or H. CD45.2^{+} CD8 populations from chimeric WT:WT or WT:*Pepd*$^{-/-}$ mice. Columns and lines represent means with 95% CI error bars and points represent individual mice. Data is representative of at three to six (G) or two (H) independent experiments with 3-6 mice per group. Statistical significance was determined across experiments using two-way ANOVA.
